# Intensity level for exercise training in fibromyalgia by using mathematical models

**DOI:** 10.1186/1471-2474-11-54

**Published:** 2010-03-22

**Authors:** Maria Carolina D Lemos, Valéria Valim, Eliana Zandonade, Jamil Natour

**Affiliations:** 1 Federal University of São Paulo, São Paulo, Brazil; 2 Federal University of Espírito Santo, Vitória, Brazil

## Abstract

**Background:**

It has not been assessed before whether mathematical models described in the literature for prescriptions of exercise can be used for fibromyalgia syndrome patients. The objective of this paper was to determine how age-predicted heart rate formulas can be used with fibromyalgia syndrome populations as well as to find out which mathematical models are more accurate to control exercise intensity.

**Methods:**

A total of 60 women aged 18-65 years with fibromyalgia syndrome were included; 32 were randomized to walking training at anaerobic threshold. Age-predicted formulas to maximum heart rate ("220 minus age" and "208 minus 0.7 × age") were correlated with achieved maximum heart rate (HRMax) obtained by spiroergometry. Subsequently, six mathematical models using heart rate reserve (HRR) and age-predicted HRMax formulas were studied to estimate the intensity level of exercise training corresponding to heart rate at anaerobic threshold (HRAT) obtained by spiroergometry. Linear and nonlinear regression models were used for correlations and residues analysis for the adequacy of the models.

**Results:**

Age-predicted HRMax and HRAT formulas had a good correlation with achieved heart rate obtained in spiroergometry (*r *= 0.642; *p *< 0.05). For exercise prescription in the anaerobic threshold intensity, the percentages were 52.2-60.6% HRR and 75.5-80.9% HRMax. Formulas using HRR and the achieved HRMax showed better correlation. Furthermore, the percentages of HRMax and HRR were significantly higher for the trained individuals (*p *< 0.05).

**Conclusion:**

Age-predicted formulas can be used for estimating HRMax and for exercise prescriptions in women with fibromyalgia syndrome. Karnoven's formula using heart rate achieved in ergometric test showed a better correlation. For the prescription of exercises in the threshold intensity, 52% to 60% HRR or 75% to 80% HRMax must be used in sedentary women with fibromyalgia syndrome and these values are higher and must be corrected for trained patients.

## Background

Fibromyalgia syndrome is a common painful syndrome characterized by the presence of chronic diffuse pain [1]. Patients with this syndrome have decreased physical fitness and this may influence pain intensity, and is involved in fibromyalgia syndrome pathogenesis but the question is why that should be the case [2,3]. There is evidence to suggest that physical activity modulates pain in fibromyalgia syndrome. The first investigator to observe a relationship between pain and exercise was Moldofsky [4]. He showed that deprivation of deep sleep lowered the threshold for pain in sedentary people, but failed to do so in more physically fit individuals. Patients with fibromyalgia syndrome characteristically exhibit decreased physical fitness [4]. In spite of aerobic fitness being the most important and frequently non-pharmacological intervention in the treatment of fibromyalgia syndrome [5-13], clinical trials vary a lot in relation to the prescription parameters.

Maximum oxygen uptake (VO_2_Max) is extensively used as a measure of cardiorespiratory physical fitness. It is the largest oxygen volume uptake by time unit on breathing atmospheric air during effort, and it is proportional to the product of heart output by the oxygen arteriovenous difference. The anaerobic threshold can be defined as the largest oxygen uptake (VO_2_) reached without sustained lactacidosis [14]. It can be measured either directly by lacticemia dosage or indirectly by analysis of expired gases. In this case it is called ventilatory anaerobic threshold. The aerobic-anaerobic transition may serve as a basis for individually assessing endurance performance as well as for prescribing intensities in endurance training. This model consists of two typical breakpoints that are passed during incremental exercise: the intensity at which blood lactate concentration begin to rise above baseline levels and the highest intensity at which lactate production and elimination are in equilibrium (maximal lactate steady state) [15]. Ventilatory anaerobic threshold oxygen uptake (VO_2_AT) or anaerobic threshold, although used less than VO_2_Max, is also a good physical fitness indicator, and it has the advantage of not being maximum effort dependent, i.e., it does not depend on the individual's will to cooperate, like fibromyalgia or patients with cardiovascular disease [16]. It seems to be more influenced by training than VO_2_Max and represents a safer intensity for exercise.

Training in anaerobic threshold intensity is considered moderate and corresponds to approximately 60-70% of the maximum heart rate (HRMax) in healthy people [17]. Anaerobic threshold is considered to be the gold standard to exercise prescription for healthy and well-trained people. In spite of being unfit, it was demonstrated that physical activities in the anaerobic threshold intensity is well tolerated and efficient in the fibromyalgia syndrome [12,13] and should be used to exercise prescription and to assess aerobic fitness in fibromyalgia syndrome since these patients do not achieve maximal test [2].

Spiroergometric test allows the achievement of VO_2 _and heart rate (HR) values. This is also a less invasive test to identify the anaerobic threshold. However, in clinical practice, it is not always possible to perform ergometric or spiroergometric test. Hence, mathematical formulas are widely used in exercise prescriptions [17].

For a long time, there has been a search for the formula to estimate the HRMax and with the objective of making the prescription of physical activity more feasible. Fox III (1971) formulated the HRMax predicted for the age, based on metabolic equivalents used in some activities. Perhaps because of its easy application and memorization, the formula "220 - age" was instituted as one of the most applied formulas to calculate the HRMax and the prescription of physical activity in a health population [18-22]. A meta-analysis by Robergs and Landwehr (2002) included all studies conducted until 2002 and obtained the formula "208.754 - 0.734 × age" [23]. This result is close to the model obtained for another meta-analysis in 2001: "HRMax = 208 - 0.7 × age" [24]. The Karvonen's formula [25] has been used to calculate HR of training, especially for athletes and cardiac patients [26-29]. This formula use percentage of heart rate reserve (HRR) to determine exercise intensity level (HR of training).

These mathematical models were tested in general healthy population, but not for fibromyalgia syndrome patients. Patients with fibromyalgia display dysautonomia [30-32], acute basal heart rate, and low HRMax heart rate even after being trained [12]. Taking into consideration that basal heart rate (HRB) and response to training is altered in these patients, it is necessary to assess whether these mathematical models described in the literature are suitable for prescriptions of exercise for fibromyalgia syndrome patients.

Thus, the objective of this paper was to determine how age-predicted heart rate formulas can be used with fibromyalgia syndrome populations as well as to find out which mathematical models are more accurate to control exercise intensity.

## Methods

### Patients

This is an experimental study for which the original data were obtained for reanalysis from "Aerobic Fitness Effects in Fibromyalgia" research database [12] including analysis from 60 sedentary women, from the rheumatology outpatient clinic of the Federal University of São Paulo, between 18 and 65 years of age, who met the American College of Rheumatology - ACR 90 Criteria for Classification of Fibromyalgia Syndrome [1]. Patients with cardiorespiratory diseases, cardiac symptoms, hypertension, diabetis, neurological disorders, body mass index greater than 35, hypothyroidism, or other rheumatic diseases were excluded. Physical exam including blood pressure, and basal electrocardiogram were normal. All patients were newly diagnosed and had never had previous treatment. Only acetaminophen was allowed as rescue medication. Women without regular physical training for the last 3 months were classified as sedentary.

Thirty-two patients were randomized according to the sequence arrival to supervised walking training, thrice a week, for 45 min duration for 20 weeks. The gold standard for intensity-level prescription was the heart rate at anaerobic threshold (HRAT) obtained by spiroergometric test. Heart rate was monitorized during the exercise program.

The patients were evaluated by a blinded investigator at the beginning and after 20 weeks (end of exercise program). All the patients read and signed the consent terms. The institution's Ethics Committee approved the study (protocol number 0463/05).

### Treadmill test

All the patients underwent an increasing load protocol on the treadmill, with a maximum duration of 13 min [2]. Analysis of expired gas was performed by a computerized metabolic system Vista Mini-CPX (Vacumed, USA). The spiroergometry was evaluated by a blinded investigator. The anaerobic threshold was determined by using the slope point on the curve of the oxygen ventilatory equivalent (VE/VO_2_). The HR at the end of each stage and the HRMax were monitored and recorded through a frequencymeter [12].

### Formulas Correlation

First, the HRMax estimated by "*220 - age*" and "*208 - 0.7 *× *age*" formulas were correlated with achieved HRMax obtained by spiroergometry. Specific model to fibromyalgia syndrome patients was calculated by regression analysis and was correlated with the achieved and age-predicted HRMax.

Subsequently, we obtained the ideal percentage that should be inserted into the six mathematical models to estimate the HRAT that is considered as the best parameter for intensity-level prescription. These six models used HRMax achieved at the end of the maximal effort or age-predicted HRMax and Karvonen's formula.

The models 1 and 3 used the achieved HRMax in the spiroergometric test. The models 2 and 4 used the HRMax predicted by "220 - age" formula. The models 5 and 6 used HRMax estimated by "208 - 0.7 × age" formula. Three models (1, 2, and 5) used the Karvonen's formula [24] that estimates the HR of training, also taking into account HRB: "*HR of training = HRB + % (HRMax - HRB)*".

The HRMax percentage or the HRR percentage corresponding to HRAT was identified and calculated for each mathematical model.

To evaluate whether all models can be used in fibromyalgia syndrome fit patients, the 32 individuals who underwent walking training were reassessed after 20 weeks of exercise. Data before and after training were compared.

### Statistical Analysis

The correlations between HRAT and HRMax measured by spiroergometric test and HRMax estimated by the mathematical models were studied using linear and nonlinear regression models. Residues analysis was used to verify model adequacy.

Linear models analysis (3, 4, and 6) included linear regression, Hypothesis Tests, Confidence Intervals, F-test and Student's *t*-test. Statistical significance was set at 5%. For the non linear models analysis (1, 2, and 5) and to compare all the models, the residues analysis was used and the dispersion graphs were presented showing the observed value on the Y axis and the estimated value on X axis. The Statistical Package for the Social Sciences (SPSS), releases 8.0 was used for the calculations.

## Results

Patients were homogenous to age (46 ± 11.12 years old) and body mass index (27 ± 5.02 kg/m^2^). Formulas to estimate the HRMax = 220 - age (*r *= 0.642; *p *< 0.01) and HRMax = 208 - 0.7 × age (*r *= 0.642; *p *< 0.01) had good correlation with the HRMax achieved in spiroergometric test.

**Figure 1:**
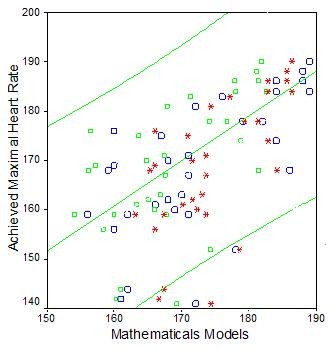
**Correlation between age-predicted HRMax *versus *the achieved HRMax values**. large circles = 220 - age, small squares = Fibromyalgia Model CI 95%, stars = 208 - 0.7 × age. HRMax = maximum heart rate, CI = confidence interval. The circles and squares are inside confidence interval (CI) of fibromyalgia syndrome formula (Fibromyalgia Model HRMax = 209 - 0.85 × age).

Specific formula to fibromyalgia syndrome patients was obtained: "HRMax = 209 - 0.85 × age". There is a good correlation between this specific formula for fibromyalgia and "220 - age" and "208 - 0.7 × age" (*r *= 0.642; *p *< 0.05) (Figure 1).

Tested mathematical models to calculate HRAT are presented in Table 1. The percentage of HRMax or the percentage of HRR corresponding to HRAT to each mathematical model is also demonstrated in Table 1.

**Table 1:** Synthesis of the tested mathematical models with ideal percentage for intensity-level, mean and standard deviation for residuals analysis.

Model	Result of Estimation	Mean	SD
1	HRAT = HRB + 60.60% (HRMax achieved - HRB)	-4.393E-02	10.02*
2	HRAT = HRB + 53.04% (HRMax^a ^- HRB)	0.2675	13.35
3	HRAT = 80.90% HRMax achieved	0.2141611	11.19
4	HRAT = 76.20% HRMax^a^	0.1594360	11.04
5	HRAT = HRB + 52.19% (HRMax^b ^- HRB)	3.015E-02	12.98
6	HRAT = 75.50% HRMax^b^	7.760E-02	13.98

HRAT = heart rate at aerobic threshold, HRB = basal heart rate, HRMax = maximum heart rate, HRMax^a ^= (220 - age); HRMax^b ^= (208 - 0.7 × age). *Lower SD indicates better model. The sequence of better models were 1>3>5>2>6>4.

There was a correlation between the achieved and estimated HRAT. All the linear models (3, 4, and 6) presented high coefficient (*R*^2 ^> 0.98, adjusted *R*^2 ^> 0.98, *p *< 0.001). All the non-linear models (1, 2, and 5) were well adjusted.

The percentage of HRMax to obtain HRAT was 80.9% for achieved HRMax, 75.5% for HRMax estimated by formula "208 - 0.7 × age" and 76.2% for the formula "220 - age". The percentage of HRR was 60.60% using achieved HRMax, 52.19% using "208 - 0.7 × age", and 53.04% using "220 - age".

Residuals analysis showed that the best adjusted mathematical model was model 1, followed by models 3, 5, 2, 6, and 4. Table 1 presents the means and standard deviations that were calculated for this analysis. The best model should have minor SD.

After training patients significantly improved fitness as demonstrated by VO_2_Max increase and VO_2 _at anaerobic threshold increase [12]. Comparisons between the models before and after the fitness program showed that HRAT was greater after fitness than before, with a statistical significance. Thus, the percentages of HRMax and HRR were higher for trained fibromyalgia women. Table 2 presents the percentages of HRMax and HRR for sedentary and fitness (after 20 weeks of training) fibromyalgia patients.

**Table 2:** Percentage used in the mathematical models to estimate intensity-level exercise in fibromyalgia.

Model	Sample	Percentage (%)	IL CI95% (%)	SL CI95% (%)
**1**	**Sedentary**	**60.60**	**57.18**	**64.02**
	**Fitness**	**74.15**	**70.69**	**77.60**
2	Sedentary	53.04	48.99	57.10
	Fitness	73.07	68.78	77.36
**3**	**Sedentary**	**80.90**	**79.30**	**82.00**
	**Fitness**	**86.00**	**84.20**	**87.00**
4	Sedentary	76.20	74.10	78.00
	Fitness	85.40	82.90	87.00
**5**	**Sedentary**	**52.19**	**48.32**	**56.06**
	**Fitness**	**71.37**	**67.28**	**75.47**
6	Sedentary	75.50	73.50	77.00
	Fitness	84.40	82.00	86.00

IL = inferior limit, SL = superior limit, CI = confidence interval, statistically different at 5%

## Discussion

This study has demonstrated that published formulas to estimate HRMax and HRR can be used for fibromyalgia syndrome patients. The validation of the published formulas to this specific population and the demonstration of the appropriate percentage to be used in these formulas to obtain intensity level at anaerobic in sedentary or fit patients with fibromyalgia will facilitate the exercise prescription in daily clinical practice by doctors, physiotherapists and physical instructors.

In order to achieve aerobic threshold intensity for training fibromyalgia syndrome patients, percentage inserted to formulas should be corrected to this population. And also, fitness fibromyalgia syndrome patients should be prescribed using higher percentages of HRMax and HRR.

Another important contribution of this study was to show which percentage to HRMax and HRR should be used in each formula to prescribe exercise on that intensity-level corresponding to HRAT. These percentages differ a little from those used in general population and suffer a lot of changes in trained patients [16] and hence, they have to be adjusted.

The achieved HRMax was better than the estimated HRMax, because it is more reliable, while HR is individual and influenced by aerobic fitness, pain intensity, and dysautonomia [33]. This study has also demonstrated that HRR is superior when compared with HRMax to indicate HR of training. Hence, HRB rate seems to be important in fibromyalgia syndrome exercise prescription. In fibromyalgia syndrome, the HRB was observed to be higher when compared with normal sedentary even in healthy subjects [13].

Age-predicted HRMax also showed good results, but "208 - 0.7 × age" formula [24] was better than "220 - age" formula [19], probably because the first was obtained in meta-analysis. Age is an important factor determining the formula composition, and it is a prediction factor to HRMax, HRB, and HRAT [34,35].

This study demonstrated that the intensity level corresponding to HRAT was 52-60% HRR and 75-80% HRMax. According previous studies the intensity level for exercise training was 60% HRR and 70-85% HRMax [21,26,27,36-39].

Obtained percentages are higher than the American College of Sports Medicine recommendation (40-49% HRR and 55-64% HRMax) to sedentary [17]. This may be owing to the fact that patients with fibromyalgia do not take maximum effort and HRmax is underestimated. In other words, HRAT is closer to the achieved HRMax in the test, when compared with the normal subjects.

This study demonstrated that the prescription percentages using achieved HRMax by spiroergometry were higher than the age-predicted HRMax formulas ("220 - age" and "208 - 0.7 × age"). According to a Brazilian research on the comparison between the intensity-level training through ergometric test, spiroergometric test, and age-predicted HRMax in healthy population, the prescriptions using formulas were observed to overestimate HRAT [40].

The majority of studies about exercise training in fibromyalgia syndrome do not describe details of prescriptions [41]. Some have used percentage of HRmax obtained by ergometry [7,42], and others have evaluated a 6 min walking test [9,43,44]. Few have used spiroergometry to detect anaerobic threshold [12,13].

Although fibromyalgia syndrome patients improved VO_2_Max, VO_2_AT, and HRAT after aerobic exercise, they hardly improved HRMax [12,13]. This effect could be explained by dysautonomia presented in fibromyalgia syndrome. In this study, it was observed higher HRAT was observed after 20 weeks, evidencing that exercises improved HRAT and percentage prescription for the same HRMax.

Meta-analysis demonstrated that the intensity-level training at anaerobic threshold or higher is necessary to improve exercise effects, especially in healthy subjects. Major changes were observed to take place close to 8-12 weeks after training, and it may be soon lost whenever training intensity is decreased [45]. Similarly, this study demonstrated comparable results. To improve aerobic fitness in aerobic-conditioned fibromyalgia syndrome patients, higher prescription percentages should be used in the formulas.

The determinations of anaerobic threshold by means of HR curve analysis have some limitation and it is not always possible due to different response to effort of myocardial function and HR [46]. However, formulas are the most used, cheap and relevant in the clinical practice for aerobic exercise prescription.

## Conclusion

In conclusion, the assessed formulas can be used to estimate HRMax and for exercise prescriptions in women with fibromyalgia syndrome. Furthermore, Karnoven's formula using HR achieved in ergometric test showed a better correlation. For the prescription of exercises in the threshold intensity, 52-60% HRR or 75-80% HRMax must be used in sedentary women with fibromyalgia syndrome, and these values may be higher and must be corrected for trained patients.

## Competing interests

The authors declare that they have no competing interests.

## Authors' contributions

MCDL participated in the analysis and interpretation of data and drafted the manuscript. VV have made conception, design, acquisition of data. Participated in the analysis and interpretation of data, drafted the manuscript. EZ performed statiscal analysis and made contributions in the design. JN participated in its design and coordination. All authors read and approved the final manuscript.

## Pre-publication history

The pre-publication history for this paper can be accessed here:

http://www.biomedcentral.com/1471-2474/11/54/prepub
